# How do contraindications to non-opioid analgesics and opioids affect the likelihood that patients with back pain diagnoses in the primary care setting receive an opioid prescription? An observational cross-sectional study

**DOI:** 10.1186/s12875-021-01386-z

**Published:** 2021-02-20

**Authors:** Michelle S. Keller, Lyna Truong, Allison M. Mays, Jack Needleman, Mary Sue V. Heilemann, Teryl K. Nuckols

**Affiliations:** 1 Division of General Internal Medicine, Department of Medicine, Cedars-Sinai Medical Center, 8700 Beverly Blvd., Los Angeles, CA 90048 USA; 2 Department of Health Policy and Management, UCLA Fielding School of Public Health, Los Angeles, CA USA; 3 Department of Biostatistics, School of Public Health, University of Michigan, Ann Arbor, MI USA; 4 Division of Geriatrics, Department of Medicine, Cedars-Sinai Medical Center, Los Angeles, CA USA; 5 UCLA School of Nursing, Los Angeles, CA USA

**Keywords:** Opioids, Back pain, Benzodiazepines

## Abstract

**Background:**

Given the risks of opioids, clinicians are under growing pressure to treat pain with non-opioid medications. Yet non-opioid analgesics such as non-steroidal anti-inflammatory drugs (NSAIDs) have their own risks: patients with kidney disease or gastrointestinal diseases can experience serious adverse events. We examined the likelihood that patients with back pain diagnoses and contraindications to NSAIDs and opioids received an opioid prescription in primary care.

**Methods:**

We identified office visits for back pain from 2012 to 2017 and sampled the first office visit per patient per year (*N* = 24,543 visits). We created indicators reflecting contraindications for NSAIDs (kidney, liver, cardiovascular/cerebrovascular, and gastrointestinal diseases; concurrent or chronic use of anticoagulants/antiplatelets, chronic corticosteroid use) and opioids (depression, anxiety, substance use (SUD) and bipolar disorders, and concurrent benzodiazepines) and estimated four logistic regression models, with the one model including all patient visits and models 2–4 stratifying for previous opioid use. We estimated the population attributable risk for each contraindication.

**Results:**

In our model with all patients-visits, patients received an opioid prescription at 4% of visits. The predicted probability (PP) of receiving an opioid was 4% without kidney disease vs. 7% with kidney disease; marginal effect (ME): 3%; 95%CI: 1–4%). For chronic or concurrent anticoagulant/antiplatelet prescriptions, the PPs were 4% vs. 6% (ME: 2%; 95%CI: 1–3%). For concurrent benzodiazepines, the PPs were 4% vs. 11% (ME: 7%, 95%CI: 5–9%) and for SUD, the PPs were 4% vs. 5% (ME: 1%, 95%CI: 0–3%). For the model including patients with previous long-term opioid use, the PPs for concurrent benzodiazepines were 25% vs. 24% (ME: -1%; 95%CI: − 18-16%). The population attributable risk (PAR) for NSAID and opioid contraindications was small. For kidney disease, the PAR was 0.16% (95%CI: 0.08–0.23%), 0.44% (95%CI: 0.30–0.58%) for anticoagulants and antiplatelets, 0.13% for substance use (95%CI: 0.03–0.22%) and 0.20% for concurrent benzodiazepine use (95%CI: 0.13–0.26%).

**Conclusions:**

Patients with diagnoses of kidney disease and concurrent use of anticoagulants/antiplatelet medications had a higher probability of receiving an opioid prescription at a primary care visit for low back pain, but these conditions do not explain a large proportion of the opioid prescriptions.

## Background

Given the well-reported serious risks of opioid therapy [1], including risk of overdose [2], opioid-related hospitalization [3], death [4], and falls and fall-related injuries [5], primary care clinicians are under growing pressure to treat pain with non-opioid medications. Comorbidities such as behavioral health disorders may place patients taking opioids at greater risk for opioid-related overdose or substance use disorders [6]. Yet non-opioid medications have their own risks, particularly among individuals with certain comorbidities and older adults [7, 8]. Many individuals are unable to use opioid alternatives such as non-steroidal anti-inflammatory drugs (NSAIDs) or other analgesics due to comorbidities such as kidney disease or gastrointestinal conditions or drug-drug interactions from concurrently prescribed medications [8]. To date, however, few studies have examined patient comorbidities associated with contraindications of non-opioid analgesics [9, 10]. Understanding opioid and non-opioid prescription patterns with regards to pain management for lower back pain can help focus quality improvement efforts and improve the development of more nuanced quality measures [7].

Our objective was to examine whether patients with contraindications for non-opioid analgesics such as NSAIDs had a higher predicted probability of receiving an opioid prescription during an office visit for low back pain. We were also interested in whether patients with comorbidities or concurrent prescriptions that place them at higher risk for overdose or substance use disorders – relative contraindications for opioids – had lower predicted probabilities of receiving an opioid prescription. We selected to study patients with a low back diagnosis given that there is extensive literature documenting that opioids are often not recommended for this condition, particularly among patients with behavioral health disorders or who are at risk for substance use disorders [11, 12]. In the U.S. workers’ compensation literature, studies have found that opioids do not expedite return to work [13, 14]. A recent Cochrane review examining the effectiveness of opioids compared to placebo or other treatments for chronic low back pain found weak evidence of the short-term efficacy of opioids compared to placebo for pain, with most trials having high drop-out rates, short durations, or showing poor improvement in function [15]. The authors noted that there is little evidence of the effectiveness of long-term opioid therapy for chronic low back pain [15].

We hypothesized that individuals with comorbidities or concurrent medication use contraindicated with NSAIDs would result in the individual having a higher predicted probability of receiving an opioid prescription, compared to individuals without these comorbidities/concurrent prescriptions, and that individuals with comorbidities and medication use associated with higher risk of overdose or opioid use disorder would have lower predicted probabilities of receiving an opioid prescription.

## Methods

### Study setting, population, and cross-sectional study design

Using administrative data, we created a dataset of patients with outpatient visits at a large, tertiary care academic health system and its associated primary care clinics. The health system is located in a metropolitan, urban area. Our population sample is primarily insured; most patients have private insurance or Medicare. In 2017, 42% of the entire population of patients seen at primary and specialty care clinic visits at the medical center had Medicare as their primary payor, 5% had Medicaid, 49% had private insurance, and 3% had a payor classified as “other” [16].

We included all patients with lower back pain diagnoses that had at least one office visit in any year between 2012 and 2017 and then extracted a year’s worth of retrospective data to identify factors associated with receipt of an opioid prescription made during the single primary care office visit. Our unit of analysis was the visit. We followed the STROBE guidelines for cross-sectional studies.

To construct the dataset, we first identified all outpatient office visits for low back pain from 2012 to 2017 for all patients seen by clinicians affiliated with the health system and sampled the first office visit per patient per year. The office visit was defined as the first time that a patient had a non-emergency, non-perioperative office visit with one of the selected ICD-9 codes identified for low back pain during each calendar year (Appendix 1). We then restricted our sample to visits to primary care clinicians. For this visit, we extracted diagnoses, prescribed medications, and demographic data. We also extracted the following data from all visits during the 365 days prior to the visit: opioid prescriptions; prescriptions of anticoagulants, antiplatelets, systemic steroids, NSAIDs; diagnoses where NSAIDs are contraindicated; and diagnoses where opioids are contraindicated (see Appendices 2 and 3 for a list of specific diagnoses and ICD-9 codes). All data were extracted from a database (Clarity) with electronic health record (EHR) data of the academic medical center.

We excluded patients under age 18 at the time of office visit, patients with a cancer diagnosis during the study period, patients pregnant during the sampled office visit, patients receiving palliative care, and individuals with vertebral fractures. These patients have specialized analgesic needs and we felt they fell outside the scope of this study. Our final sample size was 24,543 visits of patients with a low back pain diagnosis among 147 providers. The mean number of primary care office visits per patient per year was 3.6, the standard deviation was 2.6, and the range was 1–51. In the full sample, the 24,543 primary care office visits represents 17,938 unique patients over six years (2012–2017); 39.8% of patients had 1 visit represented in the sample, 27.9% had 2 visits, 16.3% had 3 visits, 8.8% had four visits, 5.2% had five visits, and 2.2% had six visits.

### Measures

#### Receipt of opioid during index visit for low back pain

The primary outcome was defined as receipt of an opioid prescription during the primary care office visit for low back pain (yes/no).

#### Comorbidities and medication use for which use of NSAIDs or opioids are contraindicated

We created separate indicators for the presence of comorbidities that have contraindications for NSAIDs, including kidney, liver, gastrointestinal, cardiovascular, and cerebrovascular diseases during the office visit or in the previous 365 days using ICD-9 codes (See Appendix 2 for a list of all of the codes used in this analysis).

Individuals may also be taking certain medications which may interact with NSAIDs or should not be prescribed concurrently due to gastrointestinal adverse effects [17–19]. These medications include long-term aspirin use, anticoagulant use, antiplatelet use, and long-term systemic steroid use [20–22]. We used medications prescribed and ICD-9 codes for long-term use of these medications in the previous 365 days before the office visit to create indicators for each of these medications (Appendix 3). We constructed categories of chronic use of these medications by counting the number of prescriptions before the office visit; if the patient had five or more prescriptions for one of these medication classes, we defined that individual as having a chronic prescription. We created one combined indicator for chronic and concurrent anticoagulant and antiplatelet use. We created one indicator each for chronic NSAID use and systemic steroid use.

We created indicators for behavioral health diagnoses, including depression, anxiety, bipolar, and substance use disorders, during the office visit and in encounters 365 days prior [23, 24]. We considered a benzodiazepine to be concurrently prescribed if it was prescribed at the same office visit as the opioid. We also controlled for age (age under 65, age 65 and older), sex (male, female), race (White, Black, Asian/Pacific Islander, unknown/missing), ethnicity (Hispanic, Non-Hispanic, unknown/ missing), employment status (employed, retired, disabled/never worked, not employed/unknown/missing), tobacco use (never use, ever smoker-quit, ever smoker-current), and marital status (single, married/domestic partnership/significant other, divorced/legally separated/widowed, other/unknown). As some of these variables can vary over time and some patients in our sample had multiple visits, we used data from the time of the sampled visit.

#### Previous opioid use

We created three categories of previous opioid use documented in the EHR:

1. no known opioid use prior to the office visit or no opioid use in the 45 days prior to the office visit;
2. intermittent opioid use, defined as use 45 days or fewer prior to the office visit but not on long-term opioids, and
3. long-term opioid use, defined as 60 or more opioid days in the 90 days prior to the office visit [25].

### Analyses

We used frequencies to examine univariate statistics and chi-square tests to examine associations between our independent and outcome variable. For our main analysis, we estimated several logistic regressions. First, we estimated a model controlling for previous opioid use, using the three categories constructed above (no opioid use in the previous 45 days, intermittent opioid use, and long-term opioid use) (Model 1). We then estimated three models (Models 2–4) stratified by previous opioid use [25]. We hypothesized that patients with prior long-term opioid use are likely to be different from patients who are opioid-naïve and who are intermittent opioid users. Patients with previous long-term opioid use may have more severe pain and also may be less affected by side effects (e.g. nausea, constipation) associated with opioids. Stratification allows the association between exposure (comorbidities and concurrent medications) and outcome (an opioid prescription at the visit) to be examined within different strata of the confounding variable (previous opioid use) [26]. For sensitivity analysis, we also controlled for the number of primary care office visits per patient per year in one model.

We controlled for the year of the prescription in all models. We used Stata version 14. We used a generalized estimating equations approach to account for multiple visits for each patient over the years and included robust standard errors to control for clustering of patients within physicians. We assumed an independent correlation matrix. We used the Stata *margins* command to estimate predicted probabilities. For continuous variables, the *margins* command calculates the predicted value of the dependent variable and then reports the mean value of those predictions. For continuous variables, Stata calculates the mean predicted value of the dependent variable if every observation in the sample had that value for the categorical variable [27]. Stata uses a bootstrap method to calculate the 95% confidence intervals [28]. We used the Stata *margins dydx* options, which estimates the marginal effect of variables [27]. Predicted probabilities can be preferrable to odds ratios as they are more intuitive and easier to understand [28]. We report predicted probabilities (i.e. margins) in the main text and odds ratios in the appendix (Appendix 5). We used the Stata-user written command *repgar* to calculate the population attributable risk using a simple logistic regression [29].

## Results

### Univariate analyses

#### Patient demographics and prior opioid use

In our model with all visits, we found that 4% of all recorded office visits for back pain resulted in a prescription for an opioid (1002 of 24,543 visits) (Table 1). Of those patient-visits where an opioid was prescribed, 47.5% of those patient-visits had at least one contraindication to NSAIDs and 52.5% did not have any documented contraindication to NSAIDs. In contrast, of the patient-visits where an opioid was not prescribed, 33.3% had at least one contraindication to NSAIDs and 66.7% did not have a contraindication to NSAIDs.

**Table 1:** Demographic and clinical characteristics of patients-visits for low back pain, 2012–2017

	No opioid prescription at office visit *n* = 23,541	Opioid prescription at office visit *n* = 1002	Total sample *n* = 24,543	***P***-Value
	N (%)	N (%)	N (%)	
**Total**	23,541 (96.9)	1002 (4.1)	24,543 (100)	
**Age**				
Under Age 65	17,674 (96.0)	739 (4.0)	18,413	
Age 65 and Older	5867 (96.0)	263 (4.0)	6130	0.45
**Sex**				
Female	14,408 (96.1)	578 (3.9)	14,986	
Male	9133 (95.6)	424 (4.4)	9557	0.03
**Hispanic Ethnicity**				
Non-Hispanic	18,463 (96.0)	811 (4.0)	19,274	
Hispanic	3762 (96.5)	137 (3.5)	3899	
Unknown/Refused	1316 (96.5)	54 (3.5)	1370	0.13
**Race**				
White	13,914 (96.0)	585 (4.0)	14,499	
Black	4427 (94.2)	274 (5.8)	4701	
Asian/Pacific Islander	2716 (98.0)	57 (2.0)	2773	
Other/Unknown	2484 (96.7)	86 (3.3)	2570	< 0.001
**Marital Status**				
Single	7399 (96.2)	296 (3.8)	7695	
Married, Domestic Partnership, or Significant Other	12,147 (96.1)	487 (3.9)	12,634	
Divorced, Legally Separated, or Widowed	3112 (94.3)	189 (5.7)	3301	
Other/Unknown	883 (96.7)	30 (3.3)	913	< 0.001
**Employment Status**				
Full Time, Self-Employed, Part-Time or Full-Time Student	14,567 (96.1)	593 (3.9)	15,160	
Retired	4304 (95.6)	199 (4.4)	4503	
Disabled or Never Worked	1048 (94.2)	64 (5.8)	1112	
Not Employed, Unknown, or Missing	3622 (96.1)	146 (3.9)	3768	0.01
**Chronic NSAID Use**				
No	23,238 (98.7)	985 (98.3)	24,223	
Yes	303 (94.7)	17 (5.3)	320	0.26
**Tobacco User**				
Never Smoker	16,493 (96.4)	609 (3.6)	17,102	
Ever Smoker, Quit	4911 (95.1)	251 (4.9)	5162	
Ever Smoker, Current	1938 (93.4)	136 (6.6)	2074	
Unknown	199 (97.1)	6 (2.9)	205	< 0.001
**Opioid Use Prior to the Index Visit**				
No Opioid Use 45 Days Prior to Index Visit	22,177 (96.8)	735 (3.2)	22,912	
Intermittent Opioid Use Prior to Index Visit	1036 (86.8)	158 (13.2)	1194	
Long-Term Opioid Use Prior to Index Visit	328 (75.1)	109 (24.9)	437	< 0.001

We found significant associations between sex, race, marital status, and employment status and receipt of an opioid prescription during the visit for low back pain in our unadjusted analyses. The proportion of patient-visits where an opioid was prescribed was higher for visits with Black patients (5.8%) and White patients (4.0%) compared to Asian/Pacific Islander patients (2.0%) and patients where the race was other or unknown (3.3%), *p* < 0.001. We did not find an association between age and receipt of an opioid prescription.

The majority of patient-visits in our sample (93.4%) had no opioid use 45 days prior to the visit. Approximately 4.9% of patients-visits were categorized as having intermittent opioid use and 1.8% of patient-visits were categorized as long-term opioid use. The proportion of patient-visits where an opioid was prescribed was higher for visits where the patient had previous long-term opioid use (24.9%) and intermittent opioid use (13.2%) compared to no opioid use recorded 45 days prior to the visit (3.2%).

### Unadjusted analyses

#### Prevalence of comorbidities and concurrent medication use for which use of NSAIDs are contraindicated

33.9% of all patient-visits had at least one comorbidity or long-term and/or concurrent prescription where NSAIDs were contraindicated. Relatively small proportions of our patient-visits had kidney disease (5.4%), liver disease (1.8%), or inflammatory bowel disease (0.8%). Higher proportions had cardiovascular or cerebrovascular disease (9.9%) and gastrointestinal disorders (10.7%). A small proportion (0.4%) had chronic systemic steroid use in the previous 365 days prior to the visit. In contrast, anticoagulant or antiplatelet use was higher: 17% of patient-visits had long-term use of these medications in the previous 365 days prior.

The proportion of patient-visits where an opioid was prescribed was higher for visits where the patient had a diagnosis of kidney disease (7.2% vs. 3.9%, *p* < 0.001), systemic steroid use, (4% vs. 4.1%, *p* = 0.002), and chronic or concurrent anticoagulant or antiplatelet use (6.9% vs. 3.5%, p < 0.001) (Table 2).

**Table 2:** Patient-visit clinical diagnoses or medication use which are contraindications for NSAIDs

	No opioid prescription at office visit *n* = 23,541	Opioid prescription at office visit *n* = 1002	Total sample *n* = 24,543	*P*-Value
	N (%)	N (%)	N	
**Kidney Disease**				
None	22,307 (96.1)	906 (3.9)	23,213	
Diagnosed	1234 (92.8)	96 (97.2)	1330	< 0.001
**Liver Disease**				
None	23,116 (95.9)	983 (4.1)	24,099	
Diagnosed	425 (95.7)	19 (4.3)	444	0.83
**Inflammatory Bowel Disease**				
None	23,357 (95.9)	991 (4.1)	24,348	
Diagnosed	184 (94.4)	11 (5.6)	195	0.27
**Cardiovascular or Cerebrovascular Disease**				
None	21,233 (96.0)	891 (4.0)	22,124	
Diagnosed	2308 (95.4)	111 (4.6)	2419	0.19
**Gastrointestinal Disorder, including GERD, Peptic Ulcers, or Bleeding**				
None	21,021 (95.9)	895 (4.1)	21,916	
Diagnosed	2520 (195.9)	107 (4.1)	2627	0.98
**Chronic Systemic Steroid Use**				
No	23,445 (95.9)	998 (4.1)	24,443	
Yes	96 (96.0)	4 (74.5)	100	0.002
**Concurrent of Chronic Anticoagulant or Antiplatelet Use**				
No	19,662 (96.5)	713 (3.5)	20,375	
Yes	3879 (93.1)	289 (6.9)	4168	< 0.001

*Abbreviations*: *NSAIDs* Non-steroidal anti-inflammatory drugs

#### Comorbidities and medication use for which use of opioids are contraindicated

Nearly one third of our sample (25%) had at least one comorbidity or concurrent medication considered a relative contraindication for opioids (Table 3): 10% of patients-visits had a depression disorder diagnosis, 13.8% had an anxiety disorder diagnosis, and 6.1% had a substance use diagnosis. 2.6% of patients-visits were prescribed a benzodiazepine at the office visit.

**Table 3:** Patient-visit clinical diagnoses or medication use which are relative contraindications for opioids

	No opioid prescription at office visit n = 23,541	Opioid prescription at office visit n = 1002	Total sample n = 24,543	*P*-Value
	N (%)	N (%)	N (%)	
**Depression Disorder**				
No	21,206 (96.0)	876 (4.0)	22,082	
Yes	2335 (9.9)	126 (12.6)	2461	< 0.01
**Anxiety Disorder**				
No	20,333 (96.1)	830 (3.9)	21,163	
Yes	3208 (94.9)	172 (5.1)	3380	< 0.01
**Substance Use Disorder**				
No	22,168 (96.2)	875 (3.8)	23,043	
Yes	1373 (91.5)	127 (8.5)	1500	< 0.001
**Bipolar Disorder**				
No	23,275 (96.0)	975 (4.0)	24,250	
es	266 (90.8)	27 (9.2)	293	< 0.001
**Benzodiazepine Prescribed at Index Visit**				
No	22,989 (96.1)	925 (3.9)	23,914	
es	552 (87.8)	77 (12.2)	629	< 0.001

The proportion of patient-visits where an opioid was prescribed was higher for visits where the patient had a diagnosis of depression (12.6% vs. 4.0%, *p* < 0.001), anxiety, (5.1% vs. 3.9%, p < 0.001), substance use disorder (8.5% vs. 3.8%, p < 0.001), bipolar disorder (9.2% vs. 4.0%, p < 0.001), a concurrent benzodiazepine prescription (12.2% vs. 3.9%, p < 0.001) or was a current tobacco smoker (Table 3).

### Regression analyses

#### Adjusted analyses examining the associations between contraindications for NSAIDs or opioids and receipt of opioid prescription

We estimated four separate logistic regression models (Table 4): Model 1 included the full sample of visits for low back pain, adjusting for previous opioid use (*N* = 24,543). Models 2–4 were stratified according to opioid use prior to the office visit: no opioid use 45 days prior to the visit (*N* = 22,912), intermittent opioid use (*N* = 1165), or long-term opioid use (*N* = 437). All models include variables that indicate contraindications for opioids and NSAIDs. We estimated models controlling for the number of primary care office visits per year and without this variable and did not find differences between the two groups of models. We report only results from the models without this variable below.

**Table 4:** Marginal effects of clinical comorbidities and concurrent or chronic prescriptions on the probability of receiving an opioid prescription during a primary care visit for low back pain (2012–2017)

	Model 1	Model 2	Model 3	Model 4
	Full Sample ***n*** = 24,543	No Opioid Use 45 Days Prior to Index Visit ***n*** = 22,912	Intermittent Opioid Use Prior to Index Visit ***n*** = 1165	Long-Term Opioid Use Prior to Index Visit ***n*** = 437
***Contraindications to NSAIDS***				
**Kidney Disease**	0.03*** (0.01, 0.04)	0.02** (0.01, 0.04)	0.09 (0, 0.18)	−0.01 (− 0.18, 0.16)
**Liver Disease**	−0.01 (− 0.02, 0.01)	− 0.01 (− 0.03, 0.01)	− 0.05 (− 0.15, 0.05)	− 0.01 (− 0.22, 0.2)
**Inflammatory Bowel Disease**	0.01 (− 0.02, 0.04)	0.01 (− 0.02, 0.03)	0.08 (− 0.19, 0.35)	−0.03 (− 0.32, 0.25)
**Cardiovascular or Cerebrovascular Disease**	− 0.01 (− 0.01, 0)	0 (− 0.01, 0)	−0.06* (− 0.11, − 0.01)	0.01 (− 0.08, 0.1)
**Gastrointestinal Disorder, including GERD, Peptic Ulcers, or Bleeding**	−0.01 (− 0.01, 0)	−0.01 (− 0.01, 0)	−0.03 (− 0.08, 0.02)	−0.06 (− 0.16, 0.05)
**Index or Chronic Anticoagulant or Antiplatelet Use**	0.02*** (0.01, 0.03)	0.02*** (0.01, 0.03)	0.02 (−0.03, 0.07)	0.07 (−0.02, 0.16)
***Relative contraindications to opioids***				
	**Full Sample**	**No Opioid Use 45 Days Prior to Index Visit**	**Intermittent Opioid Use Prior to Index Visit**	**Long-Term Opioid Use Prior to Index Visit**
**Depression Disorder**	0 (−0.01, 0.01)	0 (−0.01, 0.01)	0 (− 0.06, 0.06)	0.01 (− 0.12, 0.13)
**Anxiety Disorder**	− 0.01 (− 0.01, 0)	0 (− 0.01, 0)	−0.07** (− 0.11, − 0.03)	0.02 (− 0.05, 0.1)
**Substance Use Disorder**	0.01*(0, 0.03)	0.02** (0.01, 0.03)	0.01 (− 0.07, 0.09)	0.03 (− 0.08, 0.15)
**Bipolar Disorder**	0.02 (− 0.01, 0.05)	0.03 (0, 0.05)	0.15* (0, 0.3)	− 0.15** (− 0.25, − 0.05)
**Chronic Systemic Steroid Use**	−0.02* (− 0.04, 0)	−0.01 (− 0.04, 0.01)	–	−0.15 (− 0.32, 0.03)
**Benzodiazepine Prescribed at Index Visit**	0.07*** (0.05, 0.09)	0.06*** (0.04, 0.08)	0.15 (−0.03, 0.33)	0.10** (0.14, 0.53)

Note: In all models, we also controlled for age, race, ethnicity, sex, marital status, employment status, tobacco use, year of the prescription, and chronic use of non-steroidal anti-inflammatory drugs. All models include variables that indicate contraindications for opioids and NSAIDs.* *p* < 0.05, ** *p* < 0.01, *** *p* < 0.001. Odds ratios and confidence intervals are available in the appendix

Having kidney disease was associated with a three-percentage-point higher probability of receiving an opioid prescription during the primary care visit for low back pain, compared to patients-visits with no kidney disease, after controlling for previous opioid use and other covariates (Model 1, marginal effect [ME]: 3%; 95% CI: 1, 4%) (Table 3). This translates to 75% greater predicted probability (PP) of receiving an opioid prescription comparing individuals with kidney disease and individuals without kidney disease (predicted probability of 7% vs. 4%) and an odds ratio of 1.78 (Appendix 5). The same positive association and similar magnitude held for those who were opioid naïve (Model 2, ME: 2, 95%CI: 1, 4%; OR: 1.76).

Having long-term or concurrent anticoagulant/antiplatelet prescription was associated with a two-percentage-point higher probability of receiving an opioid prescription during the visit, compared to patients with no such medication use, all else equal (Model 1, ME: 2, 95% CI: 1, 3%; OR: 1.70). This translates to a 50% higher probability of receiving an opioid prescription between those with long-term or concurrent anticoagulant/antiplatelet use versus those without this type of medication use (predicted probability of 6% vs. 4%). We found a similar positive association and magnitude for patients who had no prior opioid use 45 days to the visit and those with intermittent opioid use. Among intermittent and long-term users, we did not find an association between kidney disease and receipt of an opioid prescription.

Having a substance use disorder diagnosis was associated with a one-percentage-point increase in receipt of an opioid prescription at the office visit in the full sample after controlling for previous opioid use (Model 1, ME: 1, 95% CI: 0, 3%; OR 1.4) with a predicted probability of 5% vs. 4%, and a two-percentage-point increase in the opioid naïve model, with a predicted probability of 5 to 3% (Model 2, ME: 2, 95% CI: 1, 3%; OR: 1.6).

The probability of being co-prescribed a benzodiazepine was positively associated with receiving an opioid prescription during the visit across nearly all models. In our model with all patient visits, this translated to a predicted probability of receiving an opioid prescription of 11% among patients with a concurrent benzodiazepine prescription vs. 4% among patients without a concurrent prescription (Model 1, ME: 7%, 95% CI: 5, 9%; OR: 3.24). This translates to a 175% greater probability of receiving an opioid prescription between those with a concurrent benzodiazepine prescription versus those without. We had similar findings among patients with no opioid use 45 days prior to the office visit (Model 2, PP 9% vs. 3%, ME: 6, 95% CI: 4, 8%; OR: 2.9) and previous long-term use (Model 4, PP 6% vs. 2%, ME: 10, 95% CI: 14, 53%; OR: 6.2).

Interestingly, we found bipolar disorder was associated with a higher predicted probability of receipt of an opioid prescription among patients with intermittent opioid use (Model 3: PP: 28% vs. 13% ME: 15, 95% CI: 0–30%; OR: 2.9) but a significantly lower probability of receipt among patients with long-term opioid use (Model 4: PP: 10% vs. 25.5%, ME: -15, 95% CI: − 25%- -5%; OR: 0.3).

In Model 1, the population attributable risk (PAR) for NSAID contraindications was small. For kidney disease the PAR was 0.16% (95% CI: 0.08–0.23%), for anticoagulants and antiplatelets, the PAR was 0.44% (95% CI: 0.30–0.58%). For opioid contraindications, the PARs were also small: 0.13% for substance use (95% CI: 0.03–0.22%) and 0.20% for concurrent benzodiazepine use (95% CI: 0.13–0.26%).

## Discussion

In this cross-sectional study of primary care visits of patients with low back pain, we examined comorbidities and concurrent prescriptions associated with contraindications to opioid and non-opioid analgesics, finding a higher predicted probability of an opioid prescription among patients with a diagnosis of kidney disease and patients with chronic or concurrent anticoagulant or antiplatelet use compared to patients without this diagnosis or concurrent medication use. Interestingly, we found no association between age and receipt of an opioid prescription in our adjusted or unadjusted analyses; however, given that age-related medication contraindications are often kidney-related, controlling for kidney disease may have lessened the strength of that association. Likewise, we found higher predicted probabilities of receipt of an opioid prescription among patient-visits with relative contraindications for opioids, including substance use disorder and patients concurrently prescribed benzodiazepines. We found that the population attributable risk proportions were very small, thus although there is an increased likelihood of receiving an opioid prescription with these comorbidities/medications, the comorbid conditions do not explain a large proportion of opioid prescriptions.

Our models showed that the predicted probability of an opioid prescription was associated with patient-visits with kidney disease and concurrent anticoagulants or antiplatelets for opioid-naïve patient-visits, but not for long-term opioid use patient-visits. These findings may reflect more recent cautious prescribing due to the prescription opioid epidemic and release of recent opioid prescribing guidelines. For patients on long-term opioid therapy who do not have contraindications to other medications, clinicians may consider whether other pharmacological or non-pharmacological options are a better fit and might consider patient-centered tapering opioid plans.

We also found that 4% of patient-visits for low back pain received an opioid prescription. Several studies have aimed to estimate opioid prescribing patterns for low back pain in primary care. One study of 219 patients aged 20–69 years old in Washington state found that 12% of primary care patients making a first visit for low back pain received an opioid prescription [30]. Another study using data from the National Ambulatory Medical Care Survey (NAMCS) found that the percentage of office visits with an opioid prescription rose from 0.65% from 1995 to 1998 to 2.63% from 2007 to 2010 [31]. From 2007 to 2010, the authors found that 13.19% visits with a back pain diagnosis received a prescription for an opioid. Visits that included an opioid prescription for a pain complaint were more likely to have younger patients and patients without private insurance, Medicare, or Medicaid. The proportion of patient-visits for low back pain in our sample that received an opioid prescription were lower than in the nationally representative NAMCS sample. A likely explanation is that the population seen at the health system in this analysis has mostly private insurance or Medicare.

Various organizations and federal agencies are developing quality measures to examine prescribing at the system, facility, and provider levels [25, 32, 33]. These measures are aimed at assisting health system leaders in identifying variation in prescribing levels among clinicians in an effort to ultimately decrease initial opioid prescribing and long-term opioid use [25]. However, an important limitation of these measures is that they do not distinguish between patients with different types of comorbidities which reflect contraindications to NSAIDs or opioids. These quality measures may penalize clinicians, such as geriatricians, who treat a higher proportion of older patients with kidney disease or who are on anticoagulants or antiplatelet medications. Developing quality measures that incorporate patient comorbidities may more accurately capture prescribing patterns.

That patient-visits with a concurrent benzodiazepine prescription had a substantially higher probability of receiving an opioid is concerning finding because this illustrates that patients at higher risk for overdose might be receiving inappropriate opioid prescriptions [34, 35]. Similarly, we found that patient-visits with diagnosed substance use disorders had higher probabilities of receiving opioid prescriptions, although these were smaller in magnitude. While patients with a history of substance use can still be prescribed opioids if followed closely, [36] some patients can develop Opioid Use Disorders when prescribed opioid medications [37]. Health systems and provider groups might consider additional training in opioid prescribing and academic detailing to help clinicians identify patients at highest risk for Opioid Use Disorder.

In contrast to the findings of a systematic review examining the effectiveness of opioids for low back pain, which concluded that there is little evidence of the effectiveness of long-term opioid therapy for chronic low back pain [15], a recent systematic review found that non-pharmacologic therapies such as tai chi, mindfulness-based reduction, yoga, exercise, psychological therapies, multidisciplinary rehabilitation, massage, and acupuncture are effective for low back pain, although the quality of the studies was still low to moderate [38]. Serotonin-norepinephrine reuptake inhibitors (SNRIs) such as duloxetine have also been found to be effective for chronic low back pain [39]. For patients with contraindications to NSAIDs, non-pharmacological options or SNRIs may be a better choice than opioid medications, which carry important risks [1].

Our study has several limitations. Although we aimed to capture prior opioid use as accurately as possible, we may not have captured opioids prescribed outside of the health system. However, many prescribers enter recent or concurrent prescriptions into the EHR during the medication reconciliation portion of the visit, so we were able to capture prescriptions identified providers or medical staff during the visit. We may also have missing diagnoses and medications for patients if they sought care outside of the system. However, we used data from visits 365 days prior to the visit, which improves our ability to identify diagnoses and long-term medication use. We also included an extensive list of comorbidities, many of which had not been explored in papers focused on opioid prescribing. We were also likely underpowered to detect some associations among patients with long-term opioid use. Our study design did not track prescribing over time, and examined only one visit per patient per year, which limits our ability to examine prescribing decisions over multiple visits. Particularly for patients with long-term opioid use, future studies should examine rates of prescribing over time, adjusting for time-varying exposures and potential confounders. As our study provides a snapshot of prescribing in time, it is possible that some patients with visits in our intermittent opioid use and long-term opioid samples had existing prescriptions at home and were not in need of a prescription during the visit. However, as the California state medical board recommends ongoing assessment of patients receiving continuous opioid prescriptions [40] and until the widespread deployment of electronic prescribing, many patients in California had to pick up physical copies of their prescriptions [41], the majority of visits for chronic low back pain among our long-term opioid group are likely for opioid-related refills. Finally, our study data was limited to one academic medical system with a predominantly insured population in a large metropolitan area, so findings may not be generalizable to rural settings or low resource settings.

## Conclusion

In conclusion, we found that patient with diagnoses of kidney disease and concurrent use of anticoagulants and antiplatelet medications had a higher probability of receiving an opioid prescription at a primary care visit for low back pain, but these conditions do not explain a large proportion of the opioid prescriptions.

## Data Availability

The data that support the findings of this study are available on request from the corresponding author. The data are not publicly available due to federal restrictions as the data contain information that could compromise patient privacy.

## Appendix 1

Index Visit Extract Description

## Appendix 2

Comorbidities from Index Visit and Visits 365 Days Prior

## Appendix 3

Prescriptions During the Index Visit and Visits 365 Days Prior

## Appendix 4

Opioid Prescription Calculation Assumptions

## Appendix 5

Odds ratios, confidence intervals for Models 1–4.
